# A prospective natural history study protocol for clinical trial readiness in synaptic disorders

**DOI:** 10.1002/epi.70374

**Published:** 2026-07-14

**Authors:** Jillian L. McKee, Sarah M. Ruggiero, Kristin Cunningham, JoeyLynn Coyne, Ian McSalley, Michael C. Kaufman, Bintou Bane, Torrey Chisari, Jonathan Toib, Carlyn Glatts, Sarah Tefft, Julie M. Orlando, Viveknarayanan Padmanabhan, Alexander K. Gonzalez, Alicia Harrison, Charlene Woo, Stephanie A. Zbikowski, Rency Dhaduk, Johanna Mercurio, Macie McCarthy, Jan H. Magielski, Zachary Grinspan, Megan Abbott, Juliet Knowles, Hsiao‐Tuan Chao, Katherine Xiong, Elizabeth Berry‐Kravis, Sepideh Tabarestani, J. Michael Graglia, Kathryn Helde, Virginie McNamar, Charlene Son Rigby, James Goss, Scott Demarest, Andrea Miele, Benjamin Prosser, Michael J. Boland, Samuel R. Pierce, Ingo Helbig

**Affiliations:** ^1^ Division of Neurology Children's Hospital of Philadelphia Philadelphia Pennsylvania USA; ^2^ Epilepsy NeuroGenetics Initiative Children's Hospital of Philadelphia Philadelphia Pennsylvania USA; ^3^ Department of Biomedical and Health Informatics Children's Hospital of Philadelphia Philadelphia Pennsylvania USA; ^4^ Department of Neurology University of Pennsylvania Philadelphia Pennsylvania USA; ^5^ Center for Epilepsy and NeuroDevelopmental Disorders, CHOP/Penn Philadelphia Pennsylvania USA; ^6^ Division of Developmental and Behavioral Pediatrics Children's Hospital of Philadelphia Philadelphia Pennsylvania USA; ^7^ Weill Cornell Medicine New York New York USA; ^8^ Department of Pediatrics/Neurology University of Colorado and Children's Hospital Colorado Aurora Colorado USA; ^9^ Department of Neurology Stanford University Stanford California USA; ^10^ Texas Children's Hospital Houston Texas USA; ^11^ Cain Pediatric Neurology Research Foundation Laboratories, Jan and Dan Duncan Neurological Research Institute, Texas Children's Hospital Houston Texas USA; ^12^ Department of Pediatrics, Section of Neurology and Developmental Neuroscience Baylor College of Medicine Houston Texas USA; ^13^ Department of Molecular and Human Genetics Baylor College of Medicine Houston Texas USA; ^14^ Department of Neuroscience Baylor College of Medicine Houston Texas USA; ^15^ McNair Medical Institute, Robert and Janice McNair Foundation Houston Texas USA; ^16^ Department of Pediatrics, Neurological Sciences, Anatomy, and Cell Biology Rush University Medical Center Chicago Illinois USA; ^17^ Cure SYNGAP1 San Diego California USA; ^18^ STXBP1 Foundation Holly Springs North Carolina USA; ^19^ Department of Physiology University of Pennsylvania Philadelphia Pennsylvania USA

**Keywords:** clinical trial readiness, developmental and epileptic encephalopathy, epilepsy, natural history, neurogenetics, STXBP1, SYNGAP1

## Abstract

**Objective:**

*STXBP1‐*related disorder (*STXBP1*‐RD) and *SYNGAP1*‐related disorder (*SYNGAP1*‐RD) are two common genetic synaptopathies that are associated with epilepsy, developmental delay, intellectual developmental disorder, and behavioral problems. Both *STXBP1*‐RD and *SYNGAP1*‐RD are potential targets for disease‐modifying therapies, but there is limited information in the literature describing the natural history of either disorder, which impedes outcome selection for future clinical trials. The objective of this study is to develop a framework to better define and outline the clinical spectrum and longitudinal trajectories of *STXBP1*‐RD and *SYNGAP1*‐RD natural history, including development, behavior, seizure histories, and electrophysiology.

**Methods:**

Here, we describe a protocol, regulatory structure, and supportive preliminary data for multicenter, prospective natural history studies of *STXBP1*‐RD (STARR) and *SYNGAP1*‐RD (ProMMiS). The protocols incorporate gold standard clinician‐administered outcome measures including the Bayley Scales of Infant and Toddler Development 4th edition, Gross Motor Function Measure‐66, fine motor domains of the Peabody Developmental Motor Scales 3rd edition, parent‐reported outcome measures, epilepsy histories, and biomarker exploration. To date, the study has enrolled 164 individuals with *STXBP1*‐RD and 159 with *SYNGAP1*‐RD, with ongoing longitudinal assessments every 6 months in a subset of approximately 200 total individuals across both disorders.

**Results:**

Our data support that existing developmental measures are feasible, informative, and show minimal floor or ceiling effects. Furthermore, we demonstrate that medical record‐based seizure history reconstruction reveals unique epilepsy trajectories while minimizing burden to families. We observe disease‐specific patterns of developmental performance and distinct longitudinal seizure dynamics, highlighting the need for data generation in a gene/disorder‐specific manner for clinical trial readiness.

**Significance:**

In summary, we present a feasible natural history protocol with prospective data for two complex neurodevelopmental disorders with natural histories that have previously been incompletely characterized, within a regulatory framework that will support the use of these data to expedite clinical trial development.

**Key points**

- *STXBP1*‐RD and *SYNGAP1*‐RD are two common genetic causes of epilepsy, developmental delay, intellectual developmental disorder, and behavior problems.
- *STXBP1*‐RD and *SYNGAP1*‐RD are potential targets for drug and gene therapy, but there is limited information in the literature describing the natural history of either disorder, which impedes the development of possible therapeutics.
- The current paper is a description of a prospective natural history study of *STXBP1*‐RD and *SYNGAP1*‐RD that will assess the clinical spectrum of each disorder through detailed developmental assessments, seizure histories, behavioral assessments, and electronic medical record reconstruction.
- By studying *STXBP1*‐RD and *SYNGAP1*‐RD with both cross‐sectional and longitudinal assessments, we aim to improve clinical trial readiness for expeditious assessment of potential treatments.

## 1 INTRODUCTION

*STXBP1‐* and *SYNGAP1*‐related disorders (RDs) are two of the most common genetic developmental and epileptic encephalopathies (DEEs),^1^, ^2^ and both have been established as prime targets for gene‐specific therapies given the severe burden of illness and haploinsufficiency as the disease mechanism.^3^ Despite the relative prevalence of these two neurogenetic disorders, most of the scientific knowledge on clinical outcomes and seizure dynamics has been shaped by retrospective data.^4^, ^5^, ^6^ Given the urgent need to develop high‐quality, prospective natural history data for future clinical trials, we launched multisite, prospective natural history studies (NHS) for both *STXBP1*‐RD and *SYNGAP1*‐RD.

*STXBP1*‐RD and *SYNGAP1*‐RD are disorders of synaptic transmission resulting in a range of neurodevelopmental symptoms and epilepsy, with distinct phenotypic profiles. *STXBP1*‐RD is predominantly characterized by global developmental delay and severe to profound intellectual developmental disorder, motor deficits driven by hypotonia and tremor, and neurobehavioral issues such as hyperactivity, aggression, and autism.^4^, ^7^, ^8^ Approximately 80% of patients have some form of epilepsy, which most often arises in the neonatal or infantile periods, but onset has been seen as late as the teenage years.^9^, ^10^ Individuals with *STXBP1*‐RD can variably present with multiple epilepsy syndromes, including Ohtahara syndrome, West syndrome, and Lennox–Gastaut syndrome. *SYNGAP1*‐RD is characterized by global developmental delay, intellectual developmental disorder, hypotonia, and generalized epilepsy often presenting as myoclonic–atonic epilepsy, and epilepsy with eyelid myoclonia, with onset typically in the toddler years.^11^, ^12^, ^13^, ^14^, ^15^ *SYNGAP1*‐RD also presents a complex neuropsychiatric profile that can include autism spectrum disorder, sleep difficulties, sensory processing disorders, and sometimes severe behavioral challenges.^6^, ^11^ Current treatment options for *STXBP1*‐RD and *SYNGAP1*‐RD are limited and focused on symptom management. Antiseizure medications are the primary intervention for seizures, although drug resistance is common, resulting in frequent changes in medication over the lifespan.^4^, ^8^, ^16^, ^17^ Unfortunately, there are currently no drugs available to directly or indirectly address the cognitive impairments and developmental delays associated with *STXBP1*‐RD and *SYNGAP1*‐RD.

Despite increased attention on *STXBP1*‐RD and *SYNGAP1*‐RD as targets for disease‐modifying therapies, knowledge of the natural history of both disorders has been limited to retrospective phenotyping efforts and case reports.^4^, ^5^, ^6^ Seizures are a common symptom among individuals with *STXBP1‐*RD and *SYNGAP1*‐RD, but disease concept models for both disorders have demonstrated that seizures present just one of many challenges for patients and caregivers, emphasizing the urgency of considering nonseizure outcomes as potential endpoints for future interventional studies.^18^ A disease concept model for *STXBP1*‐RD found that developmental delay, behavior, and seizures were major concerns for caregivers that affected the autonomy, socialization, and school performance of their children, while impacting their emotional state, need for support, and daily life.^19^ Parents and caregivers of children with *SYNGAP1*‐RD have reported that difficulty with motor skills, delays in language development, a high rate of autism spectrum disorder, and the presence of seizures were common features of the disorder.^20^

Clinical trial readiness has become a prominent issue in the field. The advancement of precision therapies for rare neurodevelopmental disorders, such as antisense oligonucleotides and gene therapy, has rapidly expanded over the past several years, with numerous novel candidate therapies under development.^3^, ^21^, ^22^ To evaluate the potential efficacy of these treatments, a detailed understanding of the natural histories of these disorders is required, an opinion also recently emphasized by guidance from the US Food and Drug Administration (FDA).^23^ Thus, it is imperative to fully describe a disease's presentation at various timepoints and to see how the condition may change over time to fully inform inclusion criteria and outcome measure selection for future interventional trials.^24^

Here, we describe our prospective natural history protocols for *STXBP1*‐RD (STARR) and *SYNGAP1*‐RD (ProMMiS) and present initial data supportive of our study design. We aim to develop a comprehensive study to characterize the natural history of *STXBP1*‐RD and *SYNGAP1*‐RD, while facilitating the creation of an NHS framework that can be generalized to expedite clinical trial readiness in other DEEs more broadly.

## 2 MATERIALS AND METHODS

### 2.1 Study organization and management

The structure of the NHS for synaptic disorders is a prospective, multisite, noninterventional study that will characterize the natural history in participants with *STXBP1*‐RD and *SYNGAP1‐*RD (Figure 1). Children's Hospital of Philadelphia (CHOP) is the prime site and the Data Coordinating Center for both studies. For the *STXBP1‐*RD arm of the study (STARR), the sites include Baylor College of Medicine/Texas Children's Hospital, Children's Hospital Colorado, Stanford University Medical Center, Rush University Medical Center, Weill Cornell Medicine, and CHOP. For the *SYNGAP1‐*RD arm (ProMMiS), study sites include Children's Hospital Colorado, Stanford University Medical Center, and CHOP. All study sites received approval from their local institutional review boards (IRBs), and the CHOP IRB holds agreements with each site's principal investigator for regulatory oversight of respective activities.

**FIGURE 1:**
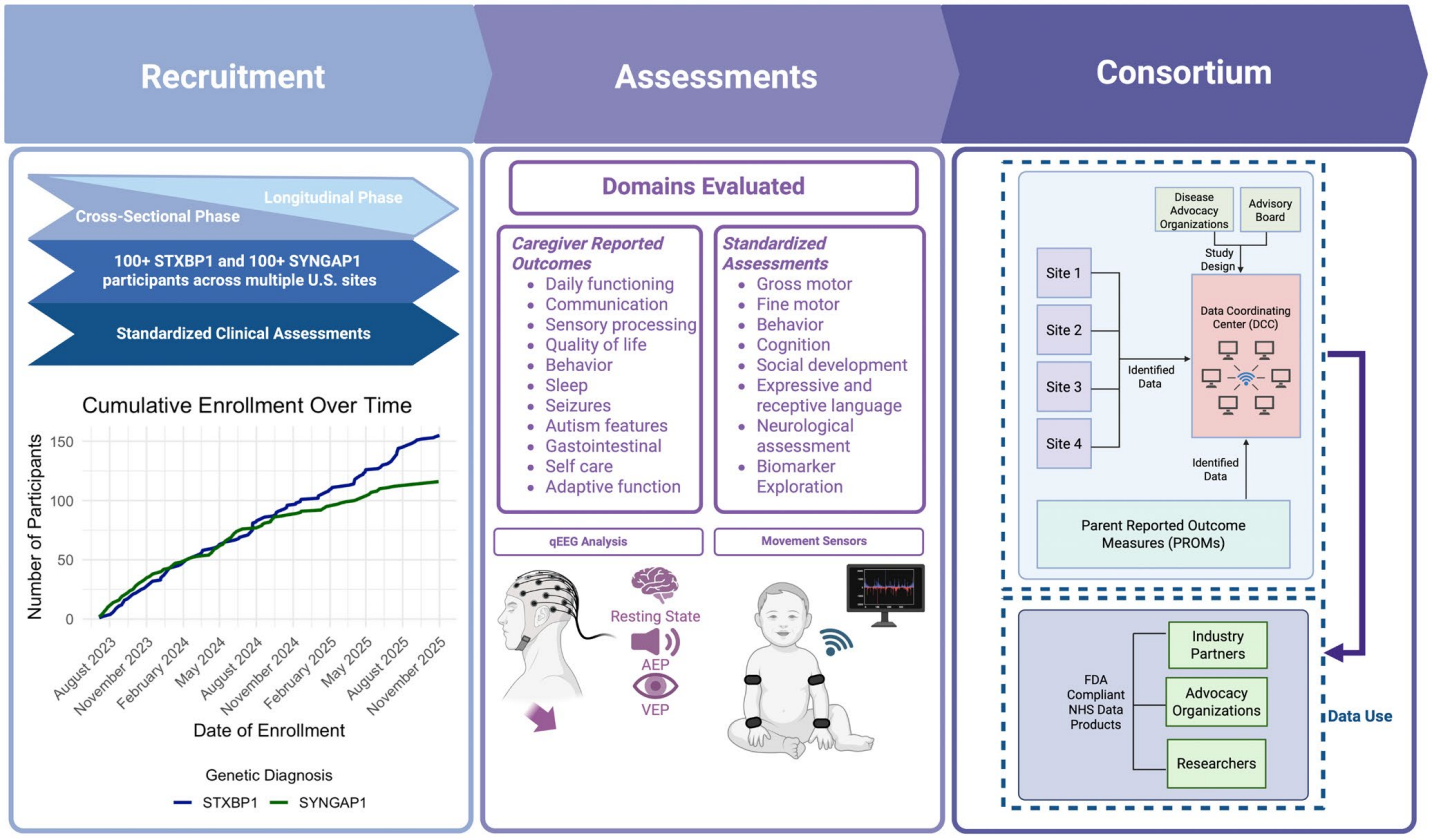
Protocol and recruitment. Here, we present a comprehensive and feasible protocol for evaluating the natural history of *STXBP1* (STARR) and *SYNGAP1* (ProMMiS) using existing outcome measures within the framework of a clinical visit. Recruitment is ongoing, with *n* = 323 individuals evaluated to date. Study protocol includes standardized clinical assessments, parent‐reported outcomes, quantitative electroencephalographic (qEEG) and movement sensors, and detailed seizure histories. Consortium structures have been established for both studies allowing data generation from multiple sites in a US Food and Drug Administration (FDA)‐compliant format for collaboration with industry partners, advocacy organizations, and researchers. AEP, auditory evoked potentials; NHS, natural history study; VEP, visual evoked potentials.

### 2.2 Study design

The NHS has two main aims: first, to cross‐sectionally evaluate as many individuals as possible to fully capture the phenotypic breadth of the disorders; and second, to follow a subset of 200 individuals longitudinally every 6 months to understand developmental trajectories and assess the stability of the measures over time. Although we encourage all participants to return every 6 months for longitudinal evaluations, we do not limit study enrollment to those committed to repeat visits. The natural history of each condition is captured alongside concurrent standard‐of‐care treatments, including but not limited to antiseizure medications, physical and occupational therapy, speech–language therapy, behavioral therapy, dietary therapy, and surgical intervention.

### 2.3 Recruitment

Subject recruitment in the ongoing NHS follows various mechanisms. Eligible subjects at each site are referred by clinicians, who may include the study investigators. Recruitment is conducted in collaboration with patient advocacy groups, and includes outreach through postings on clinicaltrials.gov, social media, and sharing of study information through family organizations. Also, patients may be referred by outside clinicians to the study or through commercial laboratories that have performed diagnostic genetic testing. Once eligible subjects are identified, their medical record is reviewed for eligibility, and the family or subject is then contacted. Parents or legal guardians of potential subjects are asked to sign a written consent before participating in the study. Due to the expected cognitive function of most children with *STXBP1*‐RD and *SYNGAP1*‐RD, a waiver for assent was obtained for the study. Adults who can provide consent or assent will sign the appropriate forms prior to participating. See Table 1 for study inclusion and exclusion criteria.

**TABLE 1:** Inclusion/exclusion criteria.

Inclusion criteria
Male or female of any age
Presence of a *STXBP1* or *SYNGAP1* causative genetic variant: ◦ Variant must be classified as causative based on clinical and variant classification as determined by a genetic reviewer ◦ Historical documentation of past result is sufficient ◦ Confirmatory testing will be obtained, if necessary, at baseline and performed by a CLIA‐certified laboratory
Exclusion criteria
Presence of a confirmed mutation in a gene other than *STXPBP1* or *SYNGAP1* that is known to contribute to a neurodevelopmental disability, including full gene deletions of *STXBP1* or *SYNGAP1* that include other genes beyond *STXBP1* or *SYNGAP1*
Presence of a significant non‐*SYNGAP1*‐RD or non‐*STXBP1*‐RD related central nervous impairment/behavioral disturbance that would confound the scientific rigor or interpretation of results of the study
History of interventricular hemorrhage or structural brain deficit
Presence of a clinical comorbidity deemed by the investigator to potentially confound the typical presentation of *SYNGAP1*‐RD or *STXBP1*‐RD
Pregnant women or females of reproductive age who are found to be pregnant upon urine pregnancy testing

Abbreviations: CLIA, Clinical Laboratory Improvement Amendments; RD, related disorders.

We have enrolled 164 individuals with *STXBP1*‐RD and 159 individuals with *SYNGAP1*‐RD to date, ranging in age from 6 weeks to 68 years. Clinical visits occur over 1 or 2 days depending on the clinical site and/or the needs of the participant. We plan to follow a subset of approximately 200 individuals longitudinally, with an initial baseline visit and follow‐up assessments every 6 months.

### 2.4 Developmental and parent‐reported outcome measures

The selection of clinical outcome measures (COMs) and parent‐reported outcome measures (PROMs) was completed in collaboration with the *STXBP1* Foundation and CURE SYNGAP1. An initial list of outcome measures was drafted to comprehensively assess relevant areas of impairment and functional limitation, as well as common medical complications associated with *STXBP1*‐RD and *SYNGAP1*‐RD. The complete list of assessments performed as part of the NHS is outlined in Table 2, and includes scales and measures selected to capture all domains of development, including gross motor, fine motor, expressive and receptive communication, cognition, behavior, sleep, adaptive function, and symptoms of autism. These tools were chosen based on a landscape analysis of NHS and clinical trial protocols for other similar DEEs (i.e., Dravet syndrome, Angelman syndrome), as well as literature review of *STXBP1*‐RD, *SYNGAP1*‐RD, and other DEEs with similar presentations. Site investigators who had clinical experience with *STXBP1*‐RD and *SYNGAP1*‐RD provided additional input. A series of meetings with the *STXBP1* Foundation and CURE SYNGAP1 reviewed the selected outcome measures and highlighted family‐identified areas of concern. A final draft of the protocol was then approved by all parties. COMs were assessed by licensed clinicians with expertise in the domain of the test, such as physical therapists for Gross Motor Function Measure‐66 (GMFM‐66) and occupational therapists for the fine motor subtests of Peabody Developmental Motor Scales 3rd edition (Peabody‐3). PROMs are administered through a third‐party platform, RARE‐X,^25^ starting from year 2 of the study once licensing agreements are finalized. We also obtained retrospective PROM data on a separate cohort of individuals with *STXBP1*‐RD and *SYNGAP1*‐RD through Simon's Searchlight for comparison with our prospective cohort.

**TABLE 2:** Outcome measures.

Assessment	Age range	Domain	Description
**Clinician outcome measures**			
Alberta Infant Motor Scale^52^	<2 years	Gross motor function	Norm‐referenced assessment developed to measure gross motor maturation in infants from birth through independent walking
Bayley Scale of Infant and Toddler Development‐4^35^	All	Gross motor Fine motor Expressive language Receptive language Cognitive	Norm‐referenced assessment designed to measure early cognitive, language, and motor development in children from the ages of 16 days to 42 months
Childhood Autism Rating Scale 2^53^	30 months+	Behavior	Behavior rating scale intended to assist in identifying children with autism including level of symptom severity
Clinical Severity Assessment (S‐CSA Clinician)^54^	All	Motor Cognition Behavior Vision Speech Autonomic function	An assessment of function and neurological impairments that was initially developed for children with *CDKL5* deficiency disorder and adapted for *STXBP1* and *SYNGAP1*
Gross Motor Function Measure‐88^55^	5 months+	Gross motor	Standardized instrument designed to measure gross motor function in children with cerebral palsy
GMFM‐66^55^	5 months+	Gross motor	Rasch analysis score from GMFM‐88 items
Peabody Developmental Motor Scales 3rd edition: Hand Manipulation and Eye‐Hand Coordination subtests^36^	All	Fine motor	A norm‐referenced test of motor development
**Classification scales**			
Communication Function Classification System^37^	2 years+	Expressive language Receptive language	Ordinal scale of expressive and receptive communication that includes verbal and nonverbal methods
Gross Motor Function Classification System Expanded & Revised^38^	All	Gross motor	Ordinal scale of gross motor function with different age bands (0–2 years, 2–4 years,4–6 years,6–12 years, 12–18 years) that was developed for children with cerebral palsy
Manual Ability Classification System^39^	4 years+	Fine motor	Ordinal scale of the ability of hand use of objects during play and activities of daily living
Mini–Manual Ability Classification System^39^	1–4 years		
**Caregiver‐reported outcome measures**			
Aberrant Behavior Checklist 2nd edition^56^	5 years+	Behavior	Assessment of problem behaviors and psychiatric symptoms
Child Behavior Checklist^57^, ^a^	1–18 years	Behavior	Assessment of emotional, behavioral, and social problems
Children's Sleep Habit Questionnaire^58^	2 years+	Sleep	Assessment of the frequency of behaviors associated with common pediatric sleep difficulties
Clinical Severity Assessment (S‐CSA Caregiver)^54^	All	Global function	Assessment of function and neurological impairments in domains of motor, cognition, behavior, vision, speech, and autonomic function
Modified Checklist for Autism in Toddlers Revised^59^, ^a^	16–30 months	Behavior	Screening assessment of behaviors related to autism spectrum disorder
Observer‐Reported Communication Ability^60^	2 years+	Communication	Assessment of expressive language, receptive language, and pragmatic communication
Pediatric Evaluation of Disability Inventory^61^	All ages	Global function	Assessment of functional skills, level of independence, and the extent of modifications required to perform functional activities
Quality of Life Inventory‐Disability^62^	All ages	Quality of life	Assessment of the domains of social health, physical health, independence, positive emotions, leisure & the outdoors, and negative emotions
Rome IV Diagnostic Questionnaire on Pediatric Functional Gastrointestinal Disorders^63^	0–18 years	GI disorders	Screening assessment for GI disorders
Sensory Profile‐2^64^	All ages	Sensory processing	Assessment of sensory processing patterns
Vineland Adaptive Behavior Scale‐3^65^	All ages	Function	Assessment of level of adaptive functioning in daily activities

Abbreviations: GI, gastrointestinal; GMFM, Gross Motor Function Measure; S‐CSA, STXBP1‐Clinical Severity Assessment.

^a^ *STXBP1*‐related disorders only.

### 2.5 Seizure history reconstruction

Seizure patterns for both disorders were documented using a standardized seizure reconstruction method previously described.^5^, ^6^, ^26^ In brief, seizure frequencies and types were extracted from patient medical records and through detailed conversations with families during initial study visits. Following enrollment in the NHS, seizure types and frequencies were reconstructed on a month‐by‐month basis, with seizure type documented using Human Phenotype Ontology (HPO) terms and seizure frequencies converted to the Pediatric Epilepsy Learning Health System (PELHS) standardized scale.^27^, ^28^ In each time bin, seizures were marked as present or absent, and the corresponding PELHS frequency was assigned, if known. If unknown, the participant's data were excluded for that month. Of note, due to the high seizure frequencies often observed in *SYNGAP1*‐RD, we developed an expanded PELHS scale, with two additional levels of 20–50 seizures per day and >50 seizures per day to better capture the variability in seizure frequency in this cohort. Given that scores represent ranges of seizure frequencies, even seizure types considered to be “uncountable” were able to be included if an approximate seizure frequency was noted in the clinical record. To date, the seizure reconstruction has only been performed for individuals enrolled at the CHOP site. Future iterations of the protocol will include an app‐based seizure diary.

### 2.6 Quantitative EEG

Resting awake‐state electroencephalographic (EEG) data are acquired using a 128‐channel Magstim EGI system and a cap‐based electrode net, recorded at a sampling frequency of 1000 Hz. The testing is performed by trained clinical research staff, and participants are seated comfortably in a quiet and dim room. Resting EEG data are recorded for 15–20 min. Records are annotated for eyes being open or closed, with any instances of sleep or excessive motion marked for eventual exclusion from the analysis. Visual evoked potentials (VEPs) and auditory evoked potentials (AEPs) are obtained during quantitative EEG sessions. For VEPs, 400 trials of a reversing black and white checkerboard are presented. For AEPs, 520 trials of 500‐Hz sinusoidal tones of 300‐ms duration are presented at 60 dB using a free‐field speaker. Distractions are minimized, and stimuli are presented at times when subjects are calm but attentive. All EEGs are downloaded from the EGI system and converted to European Data Format. To remove artifacts such as ocular movement, blinks, cardiac noise, and muscle artifact, an automated independent component analysis pipeline is applied.^29^, ^30^ All scans have a harmonic 60‐Hz notch filter applied, followed by a 1–70‐Hz bandpass filter. Data are analyzed in Python using a custom‐built pipeline to extract the power spectra and power ratios across frequency bands. Medications are tracked at each visit so the effects of various medications on EEG spectral features can be assessed.

### 2.7 Data organization and statistical analysis

All data generated from the sites are entered into a 21 CFR Part 11‐compliant REDCap database,^31^, ^32^ and data are exported and analyzed using RStudio. Figures were generated using the *ggplot2* package in R, and the protocol figure (Figure 1) was created using BioRender.

Longitudinal developmental outcomes (assessed approximately every 6 months) will be analyzed using growth‐curve models implemented as linear mixed‐effects models to estimate within‐participant change over time while accounting for within‐subject correlation and unbalanced follow‐up. Time will be modeled continuously (e.g., months since baseline), with evaluation of both linear and nonlinear change. Models will include participant‐level random effects and clinically relevant covariates (e.g., site, seizure burden, baseline severity). Missing data will be handled primarily using likelihood‐based inference from mixed models using all available observations under a missing‐at‐random assumption, with sensitivity analyses (e.g., multiple imputation) if missingness is substantial or plausibly related to clinical status. Results will be reported as estimated mean trajectories and changes over clinically relevant intervals with 95% confidence intervals, emphasizing effect sizes and precision to inform minimally detectable change for future interventional trial powering.

## 3 PRELIMINARY RESULTS

### 3.1 NHS in synaptic disorders are feasible with excellent recruitment

Since study initiation in July 2023, we have enrolled a total of 323 individuals into the combined NHS. Of the 164 individuals with *STXBP1*‐RD, 89 (54.2%) have returned for a second visit and 53 (32.3%) have had >2 assessments. A total of 159 individuals with *SYNGAP1*‐RD have been enrolled, with 73 (45.9%) returning for a second assessment and 32 (20.1%) completing >2 evaluations (Figure 1). Given ongoing recruitment, it is difficult to determine participant retention; however, 21 individuals with *STXBP1*‐RD and 34 individuals with *SYNGAP1*‐RD had baseline visits more than 1 year ago and have not returned for a second visit, suggesting they may be lost to follow‐up. We were able to recruit a large population despite the rarity of each disorder through collaboration with the *STXBP1* Foundation and CURE SYNGAP1. Recruitment has also been aided by the natural history assessments being conducted as part of a medical clinical visit; thus, families receive concurrent clinical care, teaching, and recommendations. This incentivizes participation in the studies, while at the same time reducing burden. These participants come from all over the United States and cover a broad age range, from infancy through adulthood (Figure S1).

### 3.2 Retrospective seizure reconstruction generates consistent data without burden to families

*SYNGAP1*‐RD and *STXBP1*‐RD present notable challenges in seizure tracking, given that both disorders are notorious for the presence of difficult‐to‐count or uncountable seizures, such as eyelid myoclonia in *SYNGAP1‐*RD and epileptic spasms in *STXBP1‐*RD.^1^, ^11^ In other prospective studies, collection of daily seizure counts through seizure diaries has been the traditional methodology, but significant challenges with completeness and burden on families have been noted.^33^, ^34^ Therefore, we chose to track seizure data through a real‐world data approach by reconstructing seizure types and frequencies through medical records, a method we have previously applied to other genetic epilepsies.^4^, ^5^, ^6^, ^10^, ^26^ For preliminary analysis, seizure trajectories were reconstructed in a subset of 107 individuals with *STXBP1*‐RD and 100 individuals with *SYNGAP1*‐RD in monthly time bins totaling 902.7 patient years in *STXBP1*‐RD and 950.3 patient years in *SYNGAP1*‐RD (Figure 2).

**FIGURE 2:**
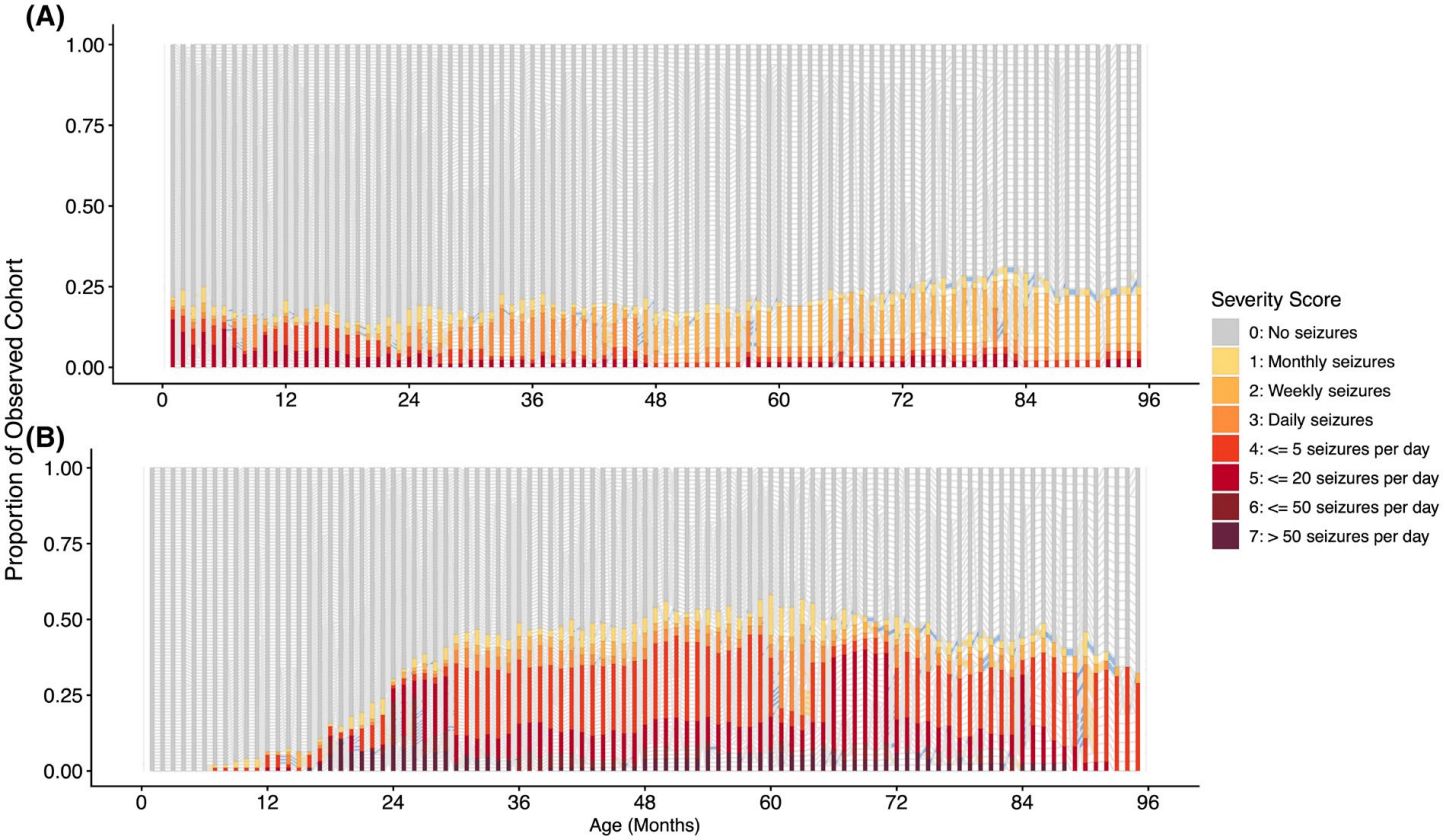
Retrospective seizure histories highlight the unique epilepsy trajectories in *STXBP1* and *SYNGAP1*. Seizure frequencies are recorded in monthly time bins from birth until the age at most recent assessment and mapped onto the expanded Pediatric Epilepsy Learning Health System frequency scale ranging from 0 (gray, no seizures) to 7 (burgundy, >50 seizures per day). Here, seizure frequency data are presented for the first 96 months of life. Yellow and blue connecting lines indicate month‐to‐month worsening or improvement in seizure frequency, respectively. This analysis illustrates the relatively stable proportion of individuals with *STXBP1*‐related disorders (A, *n* = 107) with seizures over time, with some reduction in seizure frequency as individuals age. In contrast, individuals with *SYNGAP1*‐related disorders (B, *n* = 100) tend to have overall higher rates of active epilepsy and more frequent seizures, but with later onset, with seizures typically starting in the second or third year of life.

In *STXBP1*‐RD, 73% (*n* = 78/107) of individuals experienced a seizure at any point in their lifetime, and ages at seizure onset ranged from day 0 of life through year 11.7, with a median age at onset of 2.5 months. The most frequently experienced seizure types were focal motor seizures (*n* = 61/107, 57.0%), epileptic spasms (*n* = 27/107, 25.2%), and tonic seizures (*n* = 26/107, 24.3%). At a cohort level, it was observed that within any window between 0 and 12 years of life, no more than 40% of the cohort had seizures at any one time point. In *SYNGAP1*‐RD, 84% (*n* = 84/100) of individuals had seizures at any point, with a median age at onset at 2.2 years. Most seizures were of generalized onset (78/100), with myoclonic seizures (52/100), eyelid myoclonia seizures (47/100), atonic seizures (45/100), and absence seizures (44/100) emerging as the most common types. Between the ages of 2 and 8 years, approximately 50% of the cohort was experiencing at least monthly seizures. Importantly, the frequency of seizures that could be considered countable in the context of a clinical trial was lower than the total seizure burden in both disorders. For *SYNGAP1*‐RD, countable seizures were present in 20%–45% of the cohort between the ages of 2 and 10 years and for *STXBP1*‐RD, only approximately 25% of the cohort until 10 years of age. Given countable seizures are not ubiquitously present across the cohort during target age ranges, thoughtful consideration of seizure‐related outcomes and exploration of other potential clinical trial outcome measures are essential.

### 3.3 Standardized clinical outcomes can be reliably obtained in individuals with synaptic disorders

Synaptic disorders present unique challenges in COM selection, as individuals most often present with severe global developmental delays and behavioral challenges that can make engaging in clinical assessments challenging. Creating a protocol that allows for completion in a clinic day and without significant floor or ceiling effects is paramount. We observed the appropriateness of several COMs, including the Bayley Scales of Infant and Toddler Development 4th edition (Bayley‐4), GMFM‐66,^35^ and fine motor domains of Peabody‐3.^36^ In addition, classification scales that were originally developed for children with cerebral palsy were used, including the Communication Function Classification System (CFCS),^37^ Gross Motor Function Classification System Expanded and Revised (GMFCS),^38^ Manual Ability Classification System (MACS),^39^ and Mini–Manual Ability Classification System (miniMACS).^39^ As a preliminary analysis, we assessed completion as well as floor and ceiling effects at one study site, where 89% (*n* = 87/106) of individuals with *STXBP1‐*RD were able to complete the entire protocol, with 99% (*n* = 105) of individuals being able to complete at least the GMFM‐66. Similarly, 78% (*n* = 80/116) of individuals with *SYNGAP1*‐RD assessed at baseline were able to complete the entire protocol, and 99% (*n* = 115) of individuals were able to complete at least the GMFM‐66. Analysis of the GMFM‐66 demonstrated distinct developmental patterns for both disorders, with individuals with *STXBP1*‐RD on average showing lower scores of gross motor attainment than those with *SYNGAP1*‐RD (Figure 3). Individuals unable to complete study measures were most often limited by behavior, somnolence, or travel scheduling constraints. Floor and ceiling effects, as defined by the number of individuals who achieved a minimum or maximum raw score, were negligible for *STXBP1*‐RD and *SYNGAP1*‐RD (Table S1). Classification scales including the GMFCS, CFCS, miniMACS/MACS, and a novel severity scale (STXBP1‐Clinical Severity Assessment [S‐CSA]) could be collected on all participants.

**FIGURE 3:**
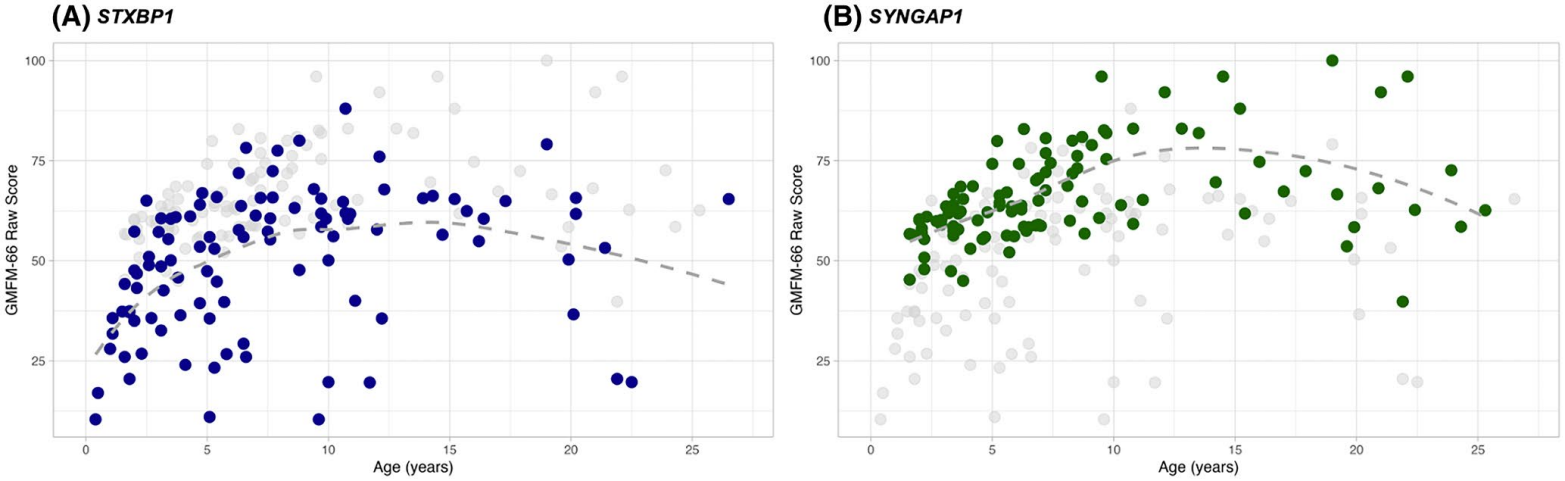
The Gross Motor Function Measure‐66 (GMFM‐66) is a feasible gross motor assessment with minimal floor or ceiling effects. Initial GMFM‐66 assessments for all individuals with *STXBP1*‐related disorders (A; blue, *n* = 105) and *SYNGAP1*‐related disorders (B; green, *n* = 115) are displayed across age. The gray dots in each plot represent the other condition to facilitate comparison. Although individuals with *SYNGAP1*‐related disorders tend to have higher gross motor function on the GMFM‐66 when compared to *STXBP1*‐related disorders, the variability in motor function of both disorders is appropriately captured by the measure, without significant floor or ceiling effects.

### 3.4 Parent‐reported outcomes can reveal disease burden not captured by clinical measures

Combining disease concept models (DCMs) for both *STXBP1*‐RD and *SYNGAP1*‐RD^18^, ^19^ with caregiver interviews and discussion with patient advocacy groups, domains of disease burden were defined and PROMs were selected to ensure coverage of all disease domains defined through the DCMs. PROMs can offer insights into disease modalities that are difficult to quantify in a clinical setting, including sleep, behavior, gastrointestinal function, and quality of life impacts, and PROMs can offer data to complement COMs. To assess feasibility of selected PROMs, we combined existing data from the Simons Searchlight database for individuals with *STXBP1* (*n* = 67) and *SYNGAP1* (*n* = 39) with the emerging data from our NHS collected using the RARE‐X platform (Figure 4). Preliminary analysis of Vineland Adaptive Behavior Scales 3rd edition suggests that this measure is appropriate and feasible. Although data are still insufficient to assess floor effects, our initial results suggest that the PROMs are feasible and can be completed by families. Further analysis is needed as our NHS accumulates data on more participants. Caregiver completion rates cannot yet be assessed; however, we are using approaches such as in‐person troubleshooting with trained staff and targeted discussion about the importance of completion with families to maximize adherence.

**FIGURE 4:**
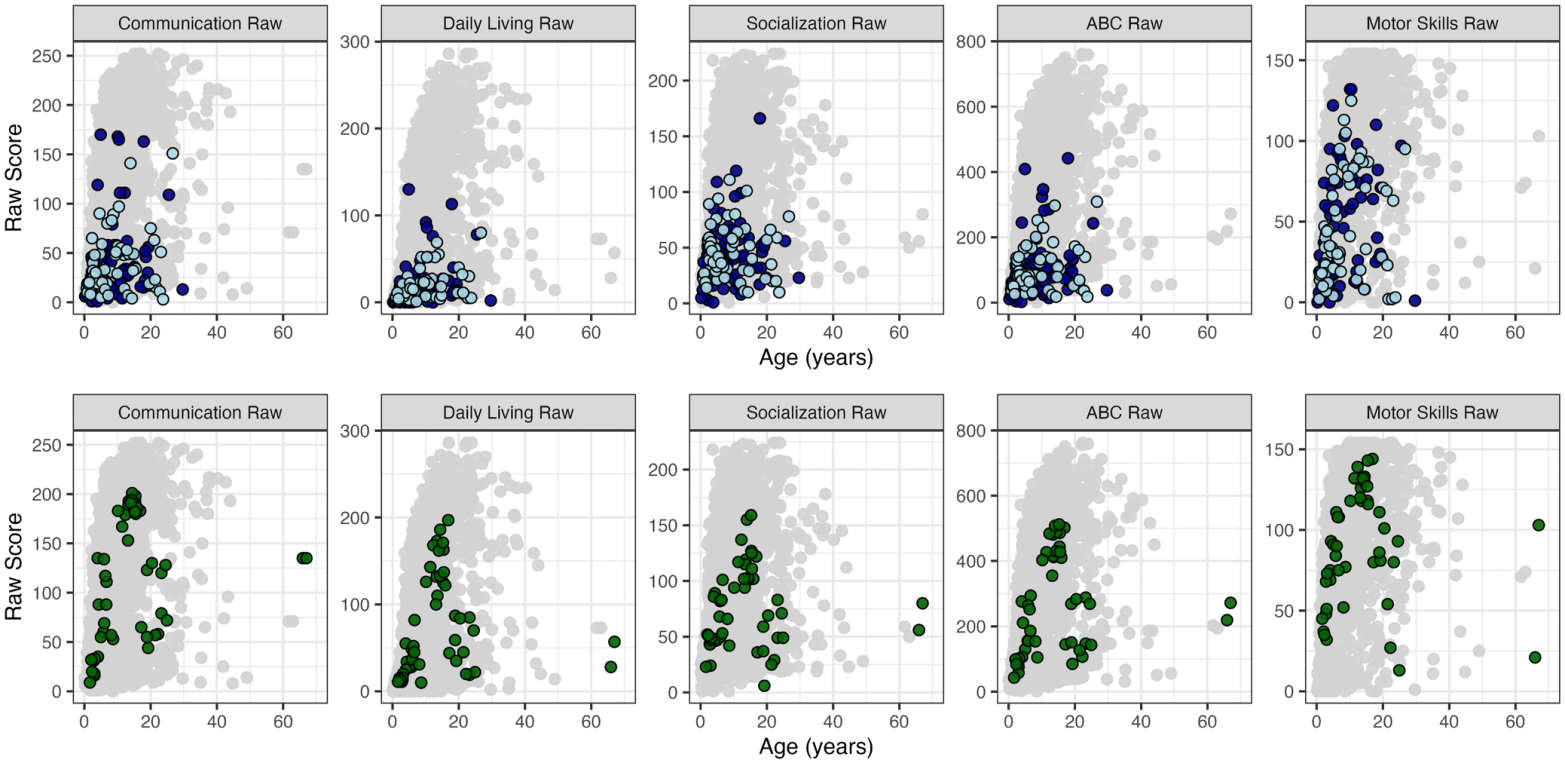
The Vineland Adaptive Behavior Scales 3rd edition (Vineland‐3) captures parent impressions of adaptive behavior across domains. Vineland‐3 data from Simons Searchlight for *STXBP1*‐related disorders (*n* = 67, top, navy) and *SYNGAP1*‐related disorders (*n* = 39, bottom, green) were compared to all others in the Simons registry (1346 individuals across 107 disorders, gray). For *STXBP1*‐related disorders, we also have preliminary data from RARE‐X for our STARR natural history study cohort, displayed in light blue (*n* = 45). The five domains are displayed separately, including communication, daily living, socialization, the ABC composite, which is a sum of the prior three scores, and motor skills. Across domains, there is a broad range of adaptive functioning, with individuals with *SYNGAP1*‐related disorders typically achieving higher performance than those with *STXBP1*‐related disorders.

### 3.5 High‐density quantitative EEGs are feasible

Resting state high‐density quantitative EEGs with visual and auditory evoked potentials are obtained using a cap‐based system every 6 months. Most participants tolerate the EEGs well, with high completion rates. To date, we have obtained 233 EEGs across 104 of 107 unique individuals with *STXBP1*‐RD and 186 EEGs across 102 of 116 individuals with *SYNGAP1*‐RD at the primary site, with each participant completing up to five repeated assessments. The main reasons studies could not be completed were participant fatigue or behavioral difficulties.

## 4 DISCUSSION

Here, we present a protocol for a comprehensive NHS for two common synaptic disorders, *STXBP1*‐RD and *SYNGAP1*‐RD, with detailed developmental, seizure, and behavioral assessments. Understanding the natural histories of both *STXBP1*‐RD and *SYNGAP1*‐RD is critical for future clinical trials that will attempt to improve the developmental trajectories, seizure frequencies, medical comorbidities, and behavioral challenges associated with these conditions. By carefully selecting the clinical assessments and PROMs for this NHS, we hope to learn detailed information regarding the appropriateness of various outcome measures for use in future clinical trials, as well as how feasible it is to collect a full battery of assessments in such complex patient populations.

Our study has three main findings. First, we demonstrate that the protocol, developed by an expert group of clinicians and patient advocates and informed by recent disease concept models, is feasible, with excellent completion rates across assessments and high participant recruitment. Second, we show through our initial data that existing outcome measures can be assessed in both *STXBP1*‐RD and *SYNGAP1*‐RD without significant floor or ceiling effects. The use of existing measures accelerates the process of obtaining natural history data and is currently favored by the FDA over the development of disease‐specific measures.^40^ Finally, our protocol is flexible, allowing for future expansion to other DEEs with similar clinical features and severity, and the use of existing standardized measures facilitates data integration with other sources and comparisons to other disorders, as well as NHS expansion and future collaborations.

The collection of clinical outcome data for individuals with *STXBP1*‐RD and *SYNGAP1*‐RD presents several challenges. Many outcome measures, such as the Bayley‐4,^35^ are norm‐referenced to children with typical development, limiting the interpretation of test results for children with *STXBP1*‐RD or *SYNGAP1*‐RD who may be older than the normative limit. As a result, the use of raw scores may be necessary, although there is concern in the literature that these values may be limited by floor effects in children with significant motor and cognitive impairments.^41^ Age equivalents are often reported in NHS,^42^ but these scores are imprecise and difficult to compare across individuals. As such, growth scale values may be preferred if available for any specific outcome measure.^43^ Reassuringly, we do not appreciate significant raw score floor effects in our preliminary data. It is also important that clinical outcome measures are sensitive to small changes, notable to families, that may result from an intervention.^44^

Due to the difficulty and time required to develop new outcome measures, the FDA has suggested that NHS use or modify existing assessments if appropriate tools are available.^40^ Here, we demonstrated the appropriateness of these existing tools in *STXBP1*‐RD and *SYNGAP1*‐RD, even when used out of age range, with no significant floor or ceiling effects noted. However, additional cross‐sectional and longitudinal data collection across all sites is necessary to gain further information about the presence of floor or celling effects in these measures. To determine whether outcome measures should be modified to more specifically assess children with *STXBP1*‐RD and *SYNGAP1*‐RD, longitudinal assessments of a large sample of children are necessary, and this will continue to be evaluated during the longitudinal portion of the NHS. In addition, further longitudinal data collection is needed to determine whether these measures are responsive to change over time. Finally, to determine whether changes in outcomes represent meaningful change to caregivers, the development of clinical global impression scales^45^ for both *STXBP1*‐RD and *SYNGAP1*‐RD is necessary to benchmark changes in outcome measures to the perceived changes that caregivers report. Because the course of development may be variable for children with *STXBP1*‐RD and *SYNGAP1*‐RD, the power to detect clinically meaningful and statistically significant differences between intervention groups necessitates careful consideration of outcome measures for a trial.

When designing the NHS, we decided to balance the competing interests of acquiring comprehensive data with minimizing the burden to families. This has resulted in several trade‐offs, most critically, the use of seizure history reconstruction rather than seizure diaries, and the requirement for in‐person assessments. In our prior work, we have demonstrated the feasibility of using retrospective medical records to reconstruct seizure trajectories in monthly time bins.^5^, ^6^ Seizure types are coded by HPO terms,^46^, ^47^ and frequencies are translated into the PELHS seizure frequency score.^27^ The preliminary seizure histories presented here are consistent with what we have previously found using the same methodology on real‐world data in both *STXBP1* and *SYNGAP1*.^5^, ^6^ As demonstrated here, seizures in *STXBP1*‐RD and *SYNGAP1*‐RD are frequently “uncountable,” challenging accurate assessments of seizure burden. As medical record data may be inaccurate and are often plagued by missing values, we plan to pilot a digital seizure application commonly used in clinical trials to validate our retrospective assessment of seizure burden in both conditions and assess the feasibility of a seizure diary for use in future clinical trials. If counting seizures is not possible, trials may need to rely on EEG‐based seizure counting or biomarkers as surrogate endpoints.

Another limitation of our study is the requirement for in‐person assessments. Given the difficulty traveling with individuals with neurodevelopmental disorders, especially the significant behavioral comorbidities associated with *SYNGAP1*‐RD, it is possible that we are limiting the participation of those who reside far from study sites or have challenging behaviors or medical needs that preclude travel. Although we cannot directly assess whether our cohort is representative of the broader population, because these are the first NHS in these conditions, previously published GMFM‐66 data obtained through clinical care on individuals with *STXBP1* are consistent with our preliminary data,^5^ and the data we present represent a range of developmental outcomes, not just mildly affected participants. To further assess the representativeness of our cohort, we plan to pilot remote use of the Developmental Assessment of Young Children^48^ in a subgroup of ProMMiS participants and validate its applicability compared to the Bayley‐4. We will then recruit additional participants who were unable to travel to a ProMMiS site and assess for any systematic differences between the cohorts. If successful, this would allow for remote participation in future clinical trials, alleviating family burden and allowing for assessment to occur in the individual's natural home environment, minimizing stress and fatigue as confounders.

Another critical component to the design of clinical trials is the identification of robust biomarkers, as these enable precise patient stratification, improve trial efficiency, and increase the likelihood of detecting true therapeutic effects.^49^, ^50^ Broadly, biomarkers are any measure that can separate individuals with a disorder from healthy controls (diagnostic), correlate with the severity of a disease (predictive), and show an improvement with disease‐modifying therapy (responsive). Our current protocol represents an initial step toward realizing the full potential of biomarker discovery. Although results were not discussed here, the NHS incorporates quantitative EEGs on all participants every 6 months, including AEPs and VEPs. Initial EEG analysis validates our prior findings that spectral features can serve as disease‐specific biomarkers in *STXBP1*‐RD and *SYNGAP1*‐RD, with reduced alpha–theta ratios in the frontal and occipital regions, respectively, compared to healthy controls.^51^ We are now evaluating the potential of event‐related potentials (AEPs and VEPs) to serve as biomarkers of disease severity. Furthermore, we are exploring the diagnostic, predictive, and responsive potential of molecular biomarkers in serum, plasma, and/or cerebrospinal fluid from individuals with *STXBP1*‐RD and *SYNGAP1*‐RD.

## 5 CONCLUSIONS

In summary, we have presented a comprehensive protocol for assessing natural history in *STXBP1*‐RD and *SYNGAP1*‐RD, including seizure trajectories, standardized developmental assessments, quantitative EEG, and PROMs assessing adaptive skills, behavior, and sleep. We additionally presented initial data demonstrating that the protocol is feasible and that individual assessments capture the full range of outcomes without significant floor or ceiling effects. Therefore, this work represents a critical first step to make *STXBP1*‐RD and *SYNGAP1*‐RD clinical trial ready.

## AUTHOR CONTRIBUTIONS

**Jillian L. McKee:** Conceptualization; investigation; formal analysis; funding acquisition; writing—original draft preparation; review & editing. **Sarah M. Ruggiero:** Conceptualization; investigation; formal analysis; project administration; writing—review & editing. **Kristin Cunningham:** Investigation; data curation. **JoeyLynn Coyne:** Data curation; project administration. **Ian McSalley:** Data curation; formal analysis. **Michael C. Kaufman:** Data curation; formal analysis. **Bintou Bane:** Data curation; project administration; writing—review & editing. **Torrey Chisari:** Data curation; project administration. **Jonathan Toib:** Data curation; formal analysis. **Carlyn Glatts:** Investigation; data curation. **Sarah Tefft:** Investigation; data curation. **Julie M. Orlando:** Investigation; data curation. **Viveknarayanan Padmanabhan:** Data curation; formal analysis. **Alexander K. Gonzalez:** Data curation; formal analysis. **Alicia Harrison:** Investigation; data curation. **Charlene Woo:** Investigation; data curation. **Stephanie A. Zbikowski:** Investigation; data curation. **Rency Dhaduk:** Data curation; project administration. **Johanna Mercurio:** Data curation; project administration. **Macie McCarthy:** Data curation; project administration. **Jan H. Magielski:** Data curation; formal analysis. **Zachary Grinspan:** Investigation; supervision. **Megan Abbott:** Investigation; supervision. **Juliet Knowles:** Investigation; supervision. **Hsiao‐Tuan Chao:** Investigation; supervision. **Katherine Xiong:** Investigation; supervision. **Elizabeth Berry‐Kravis:** Investigation; supervision. **Sepideh Tabarestani:** Investigation; supervision. **J. Michael Graglia:** Funding acquisition; project administration. **Kathryn Helde:** Funding acquisition; project administration. **Virginie McNamar:** Funding acquisition; project administration. **Charlene Son Rigby:** Funding acquisition; project administration. **James Goss:** Funding acquisition; project administration. **Scott Demarest:** Investigation; supervision. **Andrea Miele:** Investigation; supervision; writing—review & editing. **Benjamin Prosser:** Conceptualization; funding acquisition; supervision. **Michael J. Boland:** Conceptualization; funding acquisition; supervision. **Samuel R. Pierce:** Conceptualization; data curation; investigation; writing—original draft preparation. **Ingo Helbig:** Conceptualization; investigation; funding acquisition; writing—review & editing; supervision.

## FUNDING INFORMATION

This study was funded by the Center for Epilepsy and NeuroDevelopmental Disorders at Penn/CHOP; the National Institutes of Health National Institute for Neurological Disorders and Stroke (R01 NS127830 and R01 NS131512 to I.H. and K23 NS140491 to J.L.M.); the American Epilepsy Society (AES), Pediatric Epilepsy Research Foundations, and CURE SYNGAP1 through a Research Training Fellowship for Clinicians (J.L.M.); the American Academy of Neurology, AES, Epilepsy Foundation, and American Brain Foundation through the Susan Spencer Award (J.L.M.); the *STXBP1* Foundation through an individual grant (S.M.R.); and the Career Ladder Education Program for Genetic Counselors grant from the Warren Alpert Foundation (S.M.R.). Funding for investigators at Children's Hospital Colorado, Texas Children's Hospital, Rush University Medical Center, Stanford Medicine Children's Health, and Weill Cornell Medicine was provided by the *STXBP1* Foundation and for Children's Hospital Colorado and Stanford Medicine Children's Health by CURE SYNGAP1.

## CONFLICT OF INTEREST STATEMENT

H.‐T.C.'s spouse has received support from Capsida Biotherapeutics. I.H. has received support from Capsida Biotherapeutics. J.K. serves on the scientific advisory boards of CURE SYNGAP1 and the LGS Foundation and consults for BioMarin. S.D. has consulted for BioMarin, Neurogene, Marinus, Tysha, Ultragenyx, UCB, Capsida, Mahzi Therapeutics, Biogene, Praxis, Neurocrine, Alpha Rose, Azafaros, and Lundbeck. He has funding from the NIH, Project 8P, Batten Disease Support and Research Association, and *STXBP1* Foundation. He also serves on advisory boards for the nonprofit foundations SLC6A1 Connect, Project 8P, Ring14 USA, FamilieSCN2A, Inchstone, and N of 1 Collaborative. The Precision Medicine Institute at Children's Hospital Colorado has ongoing partnerships with Illumina. The remaining authors have no conflicts of interest.

## ETHICS STATEMENT

We confirm that we have read the Journal's position on issues involved in ethical publication and affirm that this report is consistent with those guidelines.

## Supporting information


FIGURE S1.
TABLE S1.

## Data Availability

The data that support the findings of this study are available on request from the corresponding author. The data are not publicly available due to privacy or ethical restrictions.
