# Supplementary material for: A prospective natural history study protocol for clinical trial readiness in synaptic disorders
Source: Epilepsia. 2026 Jul 14;67(10):5448–63. doi: 10.1002/epi.70374 (PMC13675783; doi:10.1002/epi.70374)
Supplement: Supplementary file 1 [file EPI-67-5448-s001.docx]

**Supplemental Material**

A

C

*STXBP1*

*SYNGAP1*

Age (years)

Age (years)

B

**Figure S1: Demographics.** (**A**) Geographic distribution of STARR and ProMMiS participants across the United States, illustrating the broad, nationwide recruitment of both cohorts. Histograms depict the age distributions of participants in the *STXBP1* (**B**) and *SYNGAP1* (**C**) cohorts.

|  | *SYNGAP1*-Related Disorders | | | | *STXBP1*-Related Disorders | | |
| --- | --- | --- | --- | --- | --- | --- | --- |
|  | N | Percentage Floor | Percentage Ceiling | N | | Percentage Floor | Percentage Ceiling |
| Gross Motor Function Measure-66 | 115 | 0% | 1% | 106 | | 0% | 0% |
| Peabody 3 Hand Manipulation | 94 | 0% | 2% | 87 | | 0% | 0% |
| Peabody 3 Eye Hand Coordination | 94 | 1% | 2% | 87 | | 1% | 0% |
| Bayley 4 Cognitive | 80 | 0% | 0% | 70 | | 0% | 0% |
| Bayley 4 Receptive  Language | 80 | 0% | 0% | 69 | | 0% | 0% |
| Bayley 4 Expressive Language | 81 | 0% | 6% | 69 | | 0% | 0% |
| Bayley 4 Fine Motor | 80 | 0% | 0% | 70 | | 0% | 0% |
| Bayley 4 Gross Motor | 87 | 0% | 1% | 72 | | 0% | 0% |

**Table S1: Floor and ceiling effects across measures.**
